# Current Approaches on Nurse-Performed Interventions to Prevent Healthcare-Acquired Infections: An Umbrella Review

**DOI:** 10.3390/microorganisms13020463

**Published:** 2025-02-19

**Authors:** Joana Teixeira, Neuza Reis, Ewelina Chawłowska, Paula Rocha, Barbara Czech-Szczapa, Ana Catarina Godinho, Grażyna Bączyk, João Agrelos, Krystyna Jaracz, Carlos Fontoura, Pedro Lucas, M. Rosário Pinto

**Affiliations:** 1 School of Medical and Biomedical Sciences, University of Porto (ICBAS, UP), 4050-313 Porto, Portugal; jmft@esel.pt; 2 Nursing Research Innovation and Development Centre of Lisbon (CIDNUR), 1990-096 Lisbon, Portugal; nreis@esel.pt (N.R.); paularocha@esel.pt (P.R.); anacatarinapaulos@campus.esel.pt (A.C.G.); joao.agrelos@campus.esel.pt (J.A.); prlucas@esel.pt (P.L.); 3 InfPrev4frica Project, 1600-190 Lisbon, Portugal; ewierz@ump.edu.pl (E.C.); bczechszczzapa@ump.pt (B.C.-S.); gbaczyk@ump.edu.pl (G.B.); ka.jaracz7@gmail.com (K.J.); fontoura@esel.pt (C.F.); 4 Nursing School of Lisbon (ESEL), 1600-190 Lisbon, Portugal; 5 Poznan University of Medical Sciences (PUMS), 61-701 Poznan, Poland; 6 Laboratory of International Health, Department of Preventive Medicine, 60-781 Poznan, Poland; 7 Unidade Local de Saúde Lisboa Ocidental, 1449-005 Lisbon, Portugal; 8 Epidemiology Unit, Department of Preventive Medicine, 60-781 Poznan, Poland; 9 Department of Nursing Practices, Academy of Applied Sciences in Gniezno, 62-200 Gniezno, Poland; 10 Department of Neurological Nursing, 60-806 Poznan, Poland; 11 Unidade de Investigação em Ciências da Saúde: Enfermagem—UICISA: E, 3046-851 Coimbra, Portugal

**Keywords:** hospital-acquired infection, hospital infection control, nursing care, patient safety, public health

## Abstract

To analyze nursing interventions for preventing healthcare-associated infections (HAIs), major complications in acute care impacting length of stay, costs, morbidity, and mortality, an umbrella review was conducted between 1 February and 26 February 2024, following the Joanna Briggs Institute methodology and PRISMA reporting guidelines, resulting in the inclusion of 22 articles. The 22 final articles obtained addressed the following Centers for Disease Control and Prevention (CDC) categories: surgical site infections (e.g., skin antisepsis, antibiotic prophylaxis), catheter-related bloodstream infections (e.g., taurolidine lock solutions), ventilator-associated pneumonia (e.g., oral hygiene, semi-recumbent positioning), and catheter-associated urinary tract infections (e.g., catheter duration management). Using Neuman’s holistic framework, the review emphasized patient–environment interactions. Further primary research is needed to refine these interventions and enhance interprofessional care. The protocol was registered in PROSPERO (CRD42024506801).

## 1. Introduction

Adverse events during hospital care are highly prevalent in both high-income and low- and middle-income countries. The most common are infections acquired during healthcare provision [1], the third most frequent adverse event worldwide [2,3].

Despite the positive evolution noted between the two Worldwide Reports on this subject [1,4], no nation or health system can assert that they are HAI-free [5]. According to the World Health Organization’s (WHO) Global Report on Infection Prevention and Control [1], the impact of HAIs on people’s lives is incalculable. Out of every 100 patients in acute-care hospitals, 7 in high-income countries and 15 in low–middle-income countries will acquire at least one HAI during their hospital stay. In intensive care settings, up to 30% of patients can be affected by HAIs, with an incidence that is 2 to 20 times higher in low–middle-income countries than in high-income countries, particularly among neonates [1].

Common HAI determinants are identified and associated with lack of sanitary and environmental conditions, inappropriate use of devices or antibiotics, individual conditions, poor knowledge or substandard application of infection prevention and control (IPC) guidelines [6].

WHO guidance and recommendations, developed according to the available evidence and the consensus of experts, professionals, and key stakeholders from countries and IPC fields, are available to countries at the national and facility levels [1]. International organizations regularly update guidelines to be implemented (e.g., [3,7] (ECDPC), [1,8] (WHO)).

Not only is it agreed that effective IPC programs can reduce HAI rates by 30% [5] in all known types, but a global analysis demonstrates that HAIs can be prevented by implementing evidence-based best practices, primarily by nurses [3,7,9,10,11].

However, a difference persists between the proposed measures and actual practice and real healthcare outcomes [12,13,14,15,16], as demonstrated in the statistical analysis on HAI incidence and prevalence [1,2,3,4,5,6].

Given this area’s importance for healthcare, primary and secondary studies have been conducted to explore this phenomenon. These studies produced results that contributed to the understanding of this difference. A global synthesis of the evidence available becomes important to compare published reviews (e.g., effectiveness, meta-aggregative, integrative) and thus analyze global coverage on this topic.

Therefore, an umbrella review becomes essential to examine the best existing research synthesis, enabling the analysis and sharing of findings that may contribute to the most recent guidelines released globally [17].

The Neuman Systems Model will support this analysis, as it provides a sound theoretical basis for analyzing and implementing interventions to prevent HAIs. From the perspective of this model, nursing’s objective is to reduce stressors (or the potential for stressors) by using primary, secondary, or tertiary prevention interventions aimed at maintaining, achieving, or sustaining an optimal level of well-being for the client. It is imperative for nurses to comprehensively grasp the impact of stressors, both in terms of their own impact and that of the system’s response [18,19].

The primary prevention objective entails the identification of risk factors and the prevention of stressor occurrence. Secondary prevention involves acknowledging problems and the implementation of interventions that address issues before the stressors become the target of lines of resistance. Interventions aimed at enhancing resilience include reducing or eliminating stress response and promoting equilibrium maintenance, a process which restructures the defense lines of the nurse [18,19]. This model’s comprehensive, systems-based framework enables healthcare professionals to identify and manage the complex interplay of factors contributing to HAIs and to facilitate effective prevention interventions.

## 2. Materials and Methods

We followed the Joana Briggs Institute (JBI) methodology for umbrella reviews. This strategy synthesizes the many existing systematic reviews and meta-analyses on the topic of research interest rather than conducting these systematic reviews from the ground up [17].

This manuscript complies with the PRISMA statement reporting guidelines for systematic reviews [20]. The protocol was registered in PROSPERO to ensure transparency and replicability.

We started with a preliminary search, which revealed no current or ongoing umbrella reviews on this issue.

The research question was formulated using the PI[C]O strategy [17], as our primary purpose with this review was to identify the best existing research synthesis of interventions that have shown effectiveness in preventing and controlling HAIs: Which interventions related to healthcare-associated infections (I) in hospitalized patients (P) are effective in preventing HAIs and improving patient safety (O)?

To achieve the best search strategy for each database, all identified keywords/index terms were combined with the Boolean operators OR/AND. From 1 February to 26 February 2024, the search strategy was used in the following databases: CINAHL and MEDLINE Complete, Cochrane Central Register of Controlled Trials, Cochrane Database of Systematic Reviews, JBI Evidence-Based Practice Resources on Ovid, Scopus, Web of Science and Google Scholar. The full strategy can be assessed in Appendix SA.1.

In accordance with the study’s methodology, all systematic reviews were included, excluding other study types. It was decided not to exclude studies by date due to the global impact of this health problem, with different times and perspectives in each country, aiming to ensure that we have the best evidence available about nursing interventions in a global view [16]. Language was not a limitation criterion, although the data basis descriptors used were in English.

Inclusion criteria are aligned with the research question. Regarding the Population (*P*), we encompassed studies that included both acute and hospitalized patients, making it explicit that there was not any other pre-existing kind of infection, without any increased risk of HAIs (patients in palliative care or immunocompromised) that nursing care might not be able to avert [3]. Concerning Interventions (*I*), the studies included were the ones that addressed nurse-performed interventions, including therapeutic interventions, based on their clinical judgment and expertise, to improve patient outcomes and interventions related explicitly to preventing HAIs. Studies involving interventions that nurses are unable to perform or oversee independently or in a multidisciplinary way [11] were not included. As for the outcomes (*O*), these included incidence and prevalence of HAIs, infection rates, mortality, costs, length of stay, and readmissions. Studies were excluded when other outcomes were not paired with included outcomes [3].

To extract and select the articles, the Rayyan software^®^ (https://rayyan.ai) was used in blind-on mode [21]. In addition to Rayyan software, collaborative teamwork was implemented using an internal communication platform and live-streaming meetings. After identifying and excluding duplicate entries, the screening process proceeded with selecting the study type, which JT, EC, and PR did. After this, JT, EC, PR, CG, and JA conducted the screening to verify the eligibility criteria sequentially by title, abstract and full text. All studies that met the inclusion criteria were subject to a full-text review. An independent screening validation followed the same principles, and BCS, KJ, GB, CF, and MRP ensured it. Disagreements were resolved through global group discussion, with NR and PL having the roles of arbitrators to finalize the decisions if a consensus could not be reached.

The studies’ quality was assessed according to JBI criteria for umbrella reviews [17,22,23]. The Critical Appraisal Tool for systematic reviews was used to evaluate methodological quality. The GRADE assessment was used to assess the evidence’s quality and the recommendations’ strength [24].

The synthesis of results is organized according to the CDC‘s categorization of HAIs [10], summarized in a table, and fully detailed in Appendix SA within the results section.

## 3. Results

**Figure 1:**
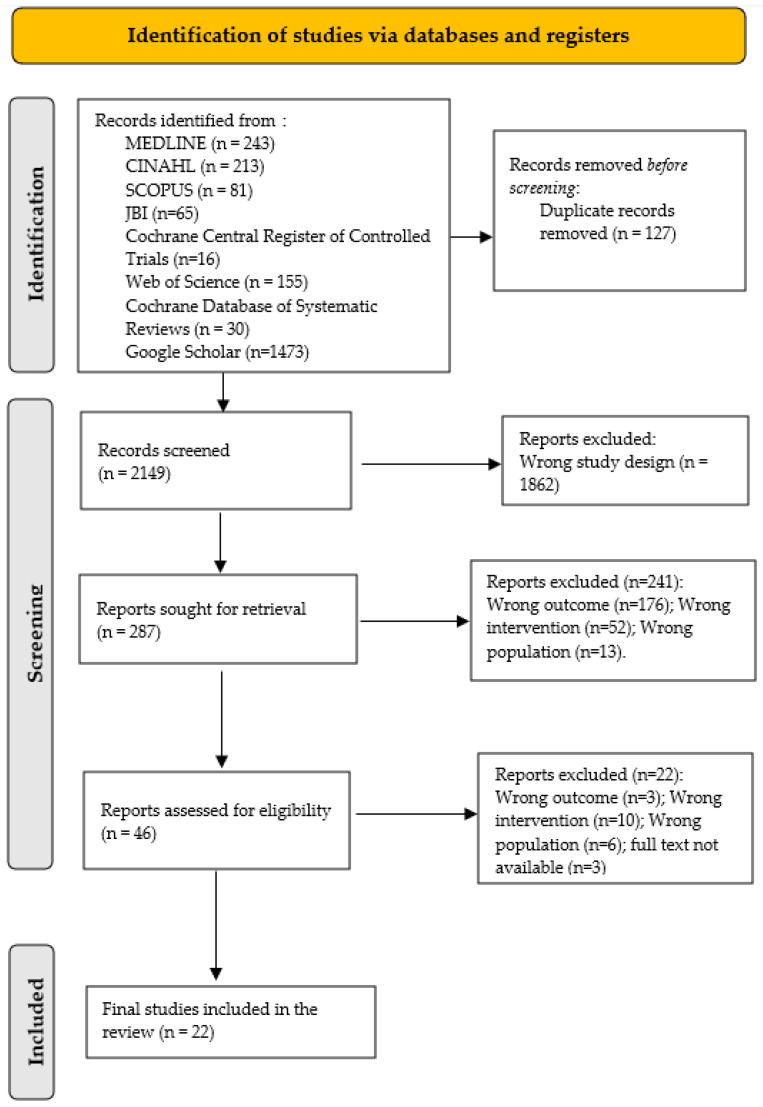
PRISMA flow diagram showing identification and inclusion process of studies [19].

After this methodological process, we included twenty-two articles; thirteen were systematic reviews [25,26,27,28,29,30,31,32,33,34,35,36,37], and nine were systematic reviews with meta-analysis [38,39,40,41,42,43,44,45,46] (see Figure 1).

Our sample includes studies published between 2006 and 2021 on four continents: America, Asia, Europe, and Oceania (Appendix SA.2).

According to the JBI quality assessment [17,23], all the studies included met a certain standard of methodological quality (Table S1).

The average quality of included studies is high (90.5%). Sixteen were high-quality studies (91–100%), and only one was low-quality (36%). In terms of evidence level and recommendation strength, the selected studies were catalogued at levels 1.a (nine) and 1.b (thirteen). The moderate GRADE rating obtained suggests moderate confidence in the effect estimate [24], related to the significant heterogeneity (Tables in Section 3).

The results’ synthesis is presented according to CDC’s Categorization of HAIs [10], summarized in Table S2, and thoroughly presented in Appendix SA.2.

### 3.1. Interventions to Improve Standard Measures to Prevent HAIs

Interventions to improve standard measures to prevent HAIs include hand hygiene, the utilization of personal protective equipment, and educational measures.

Srigley et al. [28] analyzed ten experimental and quasi-experimental studies on patient hand hygiene interventions in chronic and acute healthcare settings. These interventions aimed to improve patient hand hygiene rates and/or reduce HAIs. Most approaches mirrored the WHO multimodal strategy, incorporating education, reminders, audits, feedback, and providing hand hygiene products [28]. Results highlight that accessibility of sanitizer for patients is a key element of hand hygiene interventions for healthcare workers and patients. Their findings also show that the provision of hand antiseptics on bedside tables or food trays significantly increases patients’ hand hygiene [28]. Another major conclusion of Srigley’s team points out that directly engaging with patients results in more sustainable changes and increased adherence, especially if healthcare workers are involved in facilitating patient hand hygiene, potentiating hygiene compliance. Despite these results, Srigley and co-authors consider that the evidence obtained was low and suggest future research should be performed using stronger designs.

Still focused on hand hygiene, the review conducted by Butenko et al. [30] analyzed the relationship between healthcare professionals and patients and its effects on hand hygiene compliance. It included three qualitative studies. Results evidence that healthcare professionals recognize their role in minimizing the risk of HAIs to patients and themselves. However, the use of gloves is still identified as a perception of safety. Findings highlight that partnering for hand hygiene requires organizational and educational enablers to facilitate healthcare professional and patient partnership. Organizational enablers include appropriate equipment, sinks, trained healthcare staff, patient and staff informative documentation, posters, and educational videos. Education is recognized as a promoter of empowerment, leading patients to connect with healthcare professionals in hand hygiene performance. Despite the facilitators identified, evidence showed a lack of support for hand hygiene initiatives within the acute healthcare settings caused by the behaviors of healthcare professionals and their prevailing culture.

More focused on the partnering patient experience on hygiene compliance with healthcare professionals, Butenko et al. [30] identify that the intent to discuss and collaborate does not guarantee its translation into action. The factors influencing patients’ decision making are multifactorial and permeated with subjective and objective behavioral factors (see Table 1).

O’Horo and colleagues [45] identify infection control interventions for reducing catheter-related bloodstream infections. According to this meta-analysis, active surveillance on measures of hand hygiene and isolation precautions all contribute to preventing this type of infection.

Gavin et al. [33] reported that the highest sterile barrier measures, namely the use of large sterile drape areas; of antiseptic surgical hand rub; and the utilization of masks, caps, sterile gloves, and gowns are effective for infection prevention and control when applied during both peripheric and central catheter placement.

Educational activities and training have also been identified as impacting infection prevention. Srigley’s team [28] identifies it for hand hygiene. However, it is noted that education alone is rarely sufficient to impact long-term behavioral change. In 2008, Ramritu et al. included a pre–post intervention trial in their systematic reviews about promoting strategies in clinical practice, with a focus on prevention and staff education, stating that this could lower the risks of catheter-related bloodstream infections in the intensive care units [42].

### 3.2. Interventions for Preventing Catheter-Related Bloodstream Infections

This review comprises twelve articles that address the prevention of these infections [28,29,30,32,33,34,38,40,42,44,45,46] through various catheterization devices: periphery or central (see Table 2).

Short-term peripheric venous catheters were responsible for an average of 6.3% for nosocomial infection and 23% for catheter-related bloodstream infections [26]. Throughout this systematic review, the author identified the risk for peripheral venous catheter-related infection related to staphylococcus aureus (*S. aureus*). The results demonstrate that an increase in the risk of peripheral venous catheter-related infection is related to prolonged indwelling time along with the insertion or catheters inserted in emergent situations and that peripheral venous catheter-related infection triggered by S. aureus is significantly more probable, as well as having a significantly higher attributable mortality [26].

In light of these findings, Mermel [26] presents interventions including education and monitoring compliance to limit peripheric venous catheter dwell time to 3 or 4 days, removal of idle catheters, justification for continuous catheterization, and replacement of PVC inserted under emergent conditions, recommending daily assessment of the insertion site for localized infection or whenever a patient shows suggestive infection symptoms. Any exudate in the insertion site ought to result in the removal of the catheter and exudate culture, as evidence of localized peripheric venous catheter infection is frequently neglected. The same author points out that interventions that have been shown to minimize the incidence of peripheric venous catheter infection may not trigger the same kind of response in the incidence of phlebitis. This may happen due to chemical or mechanical irritation of the catheterized vein without the presence of infection [26].

More recently, Hopkinson et al. [29] conducted a systematic review focused on dwell time and associated complications of peripheric venous catheters. The average dwell duration was approximately three to six days, with an average of four days. Phlebitis and infiltration were the most frequent complications evaluated. Other risk factors for complications involve occlusion, catheter gauge, insertion site and type of administered infusion, along with length of hospital stay. Interventions indicated to reduce these complications include inserting catheters aseptically and keeping dwell durations under 5 days [29].

The findings of Webster et al. [31] imply that healthcare organizations should consider catheter changes only with clinical indication. To minimize complications, the insertion site should be assessed at each shift change, and catheters should be removed if there are any signs of infiltration, inflammation, or blockage or when prescribed therapy is accomplished [31].

Using catheters with antimicrobial properties may contribute to reducing the incidence of catheter-related bloodstream infections [27,29,42]. Interventions related to central vascular catheters were associated with a 57% reduction in catheter-related bloodstream infections [45]. Hocknhull et al. [37] likewise conclude that anti-infective central vascular catheters appear to be more effective in lowering catheter-related bloodstream infections than standard central vascular catheters.

In terms of central vascular catheters, Ramaritu et al. conducted a systematic review in 2008, identifying the usage of alternative solutions for skin disinfection prior to catheter insertion, the combination of Vitacuff and polymyxin ointment, neomycin, bacitracin and staff education, multifaceted infection control programs, and feedback on performance [42].

For central venous access devices, changing dressings less frequently reduces the probability of harm to the skin, although it is not clear whether this affects the rate of infections related to catheters. The association between the interval of time between central venous access device dressing changes and the frequency of catheter-related bloodstream infections is currently inconclusive [33].

However, systematic reviews by both Ho and Litton [38] and Ullman et al. [32] found that drug-impregnated dressings have a lower occurrence of catheter-related bloodstream infections compared to other types of dressing. In the 2006 review, a chlorhexidine-impregnated dressing was shown to minimize the possibility of intravascular catheter or colonization by bacteria at the exit site, as well as the risk of infection of the central nervous system or catheter-related bloodstream infections. The 2015 systematic review showed that chlorhexidine gluconate-impregnated dressings reduce catheter-related bloodstream infections when compared to standard dressings.

Still focusing on the fixation of the catheters, Ullman and his team’s meta-analysis showed that fixation without suture devices is probably the best at minimizing catheter-related bloodstream infections. However, the authors suggest that more research is needed to evaluate the effectiveness of new systems or products [32].

Regarding daily bathing with chlorhexidine gluconate, O’Horo et al.’s [45] study showed a reduction in the incidence of catheter-related bloodstream infections among patients in the medical intensive care units. However, it was impossible to draw conclusions on the recommendation based on the ideal regularity, application method, and concentration of chlorhexidine gluconate and its effectiveness compared with other preventive measures to reduce catheter-related bloodstream infections.

Using taurolidine lock solutions lowered the incidence of catheter-related bloodstream infections without apparent bacterial resistance or secondary effects [40].

In the pediatric population with central vascular catheters implanted for parenteral nutrition or drug administration, the results showed a statistically significant decrease in the number of catheter-related bloodstream infections with taurolidine compared to the control group [46].

The main results of these studies highlight that it is essential to establish whether effects remain in contexts where routine practice uses infection prevention care bundles [37] and note that while checklists are now used widely and a decline in infections has begun, in hospitals that have not yet reached very low rates of infection, additional improvements should happen, with inherent savings [27].

Furthermore, it is important to mention that nurses typically lead infection control within a broader multidisciplinary team, playing a key role in caring [42].

### 3.3. Interventions to Prevent Surgical Site Infections

Our findings on the prevention of SSI include interventions mostly focused on the utilization of antiseptics and antibiotic prophylactic management (see Table 3).

In a meta-analysis of nine randomized controlled trials (RCTs), Lee et al. [25] conclude that preoperative chlorhexidine skin antisepsis shows higher effectiveness than iodine in the prevention of SSI, with a 36% lower number of SSIs than those who received iodine.

Despite the efficiency of skin antiseptics, other sources can contaminate wounds, once the skin antiseptic has worn off [25].

The administration of antibiotic prophylaxis is a nurse-managed intervention, even if it is a medical prescription or a protocoled practice. Jonge et al. [41] conducted a systematic review and meta-analysis on the timing of preoperative antibiotic prophylaxis and the risk of SSI. Their findings show that the surgical antibiotic prophylaxis should be delivered within 120 min before the incision. In contrast to the widely established practice of providing prophylaxis within a 60 min window, no apparent advantage was discovered within the 120 min window before incision, although the generally established recommendation to provide this 60 min before incision could not be supported. However, the evidence is based on research with inadequate methodological quality, and definite randomized clinical trials are still required [41].

This team discussed more. They propose paying special attention to the assessment of the patient’s pathophysiologic circumstances. Any situation with low serum protein levels (e.g., elderly or critically ill patients) can result in suboptimal antibiotic exposure. The use of a surgical safety checklist is also recommended, since it results in higher compliance with antibiotic prophylaxis time of administration [41].

Another intervention that emerged from our umbrella review was perioperative high-inspiration oxygen. Togioka et al.’s [39] meta-analysis revealed that, generally, there is no evidence that hyperoxia is associated with lower rates of SSI. However, results from specific situations (e.g., colorectal surgery and general anesthesia) show the opposite. Other findings were analyzed in this review, including strategies for fluid management, because both hypovolemia and over-administration of crystalloids can influence SSI. Hypovolemia reduces the flow of blood to wounds, which is linked to increased infection (hypoperfusion). Furthermore, edema can prevent oxygen delivery. These authors also acknowledge the limitations of their meta-analysis, related to the differences among studies [39].

The evidence generated in this review also indicated that the use of body warming methods, and consequently the maintenance of higher body temperature averages, decrease the incidence of SSI. In the perioperative period, exposing the patient to temperatures below 36 °C increased the possibility of developing this kind of infection [43]. Nevertheless, the gaps in the knowledge produced are identified. Results suggested that the association between perioperative active warming strategies and others is still unclear.

### 3.4. Interventions to Prevent Ventilation-Associated Pneumonia (VAP)

VAP in critically ill patients is one of the main leading causes of death; additionally, it can increase hospital stay as well as healthcare costs. The position of ventilated patients while lying in bed can also play an important role in preventing lung infection [44]. Considering this, this team analyzed 10 RCTs with 878 participants to evaluate the effectiveness of a semi-recumbent position for preventing adults’ VAP associated with mechanical ventilation. Additionally, they compared the effectiveness between this position and a supine position. Wang et al.’s [44] findings state that a position between 30° and 60° significantly decreased the risk (when compared to a 0° to 10° supine position) of clinically suspected VAP (see Table 4).

However, there was no significant difference when comparing the two positions concerning the following outcomes: duration of the mechanical ventilation, microbiologically verified VAP, use of antibiotics, intensive care units and hospital mortality, and both length of stay in intensive care units and the hospital. According to the same study, there was no evidence of changes in the outcomes with statistical significance, namely the semi-recumbent position (45°), when compared to 25° to 30°. On the other hand, the balance between the possible negative and positive effects of semi-recumbent positioning is still to be confirmed due to the low number of studies and the low quality of the already gathered data [44].

Hua et al. [34] conducted a systematic review focused on evaluating the effect of oral hygiene care on the incidence of VAP in critically ill patients mechanically ventilated for at least 48 h in intensive care units, including 40 RCTs (5675 participants). These trials were categorized into five categories, grounded in intervention and co-interventions utilized, namely chlorhexidine mouth solution or gel, compared to placebo, usual care, or other oral care solutions; toothbrushing versus no toothbrushing (with or without antiseptics); and finally, powered toothbrushing and manual toothbrushing. The main conclusions from this review were that the use of chlorhexidine, both as a mouth rinse or in gel form, reduces the incidence of VAP from 26% to 18% (*p* = 0.03), a conclusion supported by moderate-certainty evidence (1206 participants, 92% adults) from 13 RCTs [34].

Endotracheal tubes seem to be an independent risk factor for VAP [35]. Nowadays, there are silver-coated endotracheal tubes that release silver cations (with antimicrobial effects), which could be used in VAP prevention [35]. According to this, Tokmaji et al. [35] conducted a review to analyze whether silver-coated endotracheal tubes can reduce the risk of VAP and hospital mortality when compared to standard non-coated endotracheal tubes, in patients under mechanical ventilation for 24 h or longer. This review included 2081 subjects divided into three eligible randomized controlled trials. The authors found that silver-coated endotracheal tubes seemed to reduce the possibility of developing VAP from 6.7% to 3.5% within 10 days of endotracheal intubation. Once more, the quality of the evidence regarding this outcome was low, with confidence intervals insufficiently narrowing to justify the effect’s magnitude [35].

### 3.5. Interventions to Prevent Catheter-Associated Urinary Tract Infections

Only one study that fulfilled inclusion criteria was identified in our research, performed in 2021. This systematic review included RCTs and quasi-RCTs that analyzed the effectiveness of the intervention taken for the removal of indwelling short-term urethral catheters. The population of the study was adults in any kind of setting catheterized for any reason, covering a total of 99 trials involving 12 241 participants [36]. These authors recognized four types of nursing intervention: removal of indwelling urethral catheters between 6 am and 7 am, compared to removal between 10 pm and midnight; shorter time lengths of indwelling urethral catheterization compared to longer time lengths of indwelling urethral catheterization; flexible durations compared to a fixed duration of indwelling urethral catheterization; and clamping compared to free drainage of indwelling urethral catheterization before removal (see Table 5).

According to the results, late-night catheter removal could decrease the risk of catheter reinsertion when compared to early-morning removal, without certain results on whether this difference would impact the development of these types of infections. The early removal of the catheters results in less frequent development of urinary tract infection and less frequent painful urination, compared with longer catheterization. However, findings identify a higher probability of needing to reinsert the catheter in cases of shorter-length catheterizations. The analysis of the clamping strategy showed little or no difference in the clamping or in keeping free drainage on the risk of urinary tract infections, painful urination, or the need for catheter reinsertion [36].

This systematic review has shown a need for a standardized set of core outcomes, and these outcomes must be measured and reported by all trials performed in the future comparing methods for the removal of these urinary catheters, suggesting that future trials should study the effects of short-term indwelling urethral catheter removal for non-surgical patients [36].

## 4. Discussion

This umbrella review reports on 374 studies, involving more than 342,453 participants, from 22 systematic reviews. Responding to the main objective of this umbrella review, an overview and appraisal of nurse-performed interventions to prevent HAIs in four key areas is provided: improving preventive standard measures, preventing catheter-related bloodstream infections, surgical site infections, VAP, and catheter-associated urinary tract infections. The contribution for preventing catheter-related bloodstream infections is broader and the most effective intervention is related to education of professionals but also of patients, depending on the type of measure under discussion.

Our results identify various levels of effectiveness across different methods. Patient and staff education, standardizing catheter care protocols, and improving hand hygiene demonstrated moderate effectiveness in reducing HAIs. Using chlorhexidine for skin antisepsis and daily bathing yielded moderate to high improvements in reducing bacterial colonization and bloodstream infections, with moderate to high certainty. Chlorhexidine-impregnated dressings and taurolidine lock solutions effectively reduce catheter-related infections, demonstrating moderate positive effects with moderate certainty. Perioperative interventions such as high oxygen therapy and warming had minor positive impacts on SSI but with low to moderate certainty. Specific interventions for VAP, such as oral hygiene and silver-coated endotracheal tubes, showed moderate positive effects.

Considering the nature of the phenomenon and the findings obtained, this chapter’s discussion integrates the existing guidelines from international organizations that can support practice worldwide in HAI prevention [3,4,5,6,7,8,9,10], using the perspective of Neuman’s Systems Model to conduct the discussion. Two main reasons justify this option. Firstly, the model’s core concepts—person, environment, health, and nursing—focus on maintaining system stability through prevention strategies. The individual is conceptualized as comprising a core or essential survival factor structure, surrounded by concentric defensive rings, and is regarded as a person, a family, a community, or a social problem. The client system develops a series of defense lines to protect environmental interaction. The normal line of defense can be interpreted as the client’s usual level of wellness [18,19]. This stability is threatened by intrapersonal, interpersonal, and extrapersonal factors (stressors). Illness occurs when these defenses fail, and well-being is restored through primary, secondary, and tertiary prevention strategies [18,19].

Using this view, HAIs are extrapersonal stressors that, by themselves or combined with intrapersonal (like physiological or development factors) or interpersonal (e.g., variability in adherence to IPC, inconsistent training, or lack of knowledge by the professionals) stressors, affect both patients and healthcare systems. Primary, secondary, and tertiary prevention strategies align with the interventions highlighted in the findings [18,19].

In our results, interventions related to standard measures focus essentially on hand hygiene for professionals, patients, and families [28], on the environmental conditions to perform this procedure [28,30,32], on the use of personal protective equipment [30,33], and the structural importance of knowledge acquisition and adherence to the available guidelines. These results are consistent with the multimodal strategy proposed by WHO, ECDC and CDC [1,3,4,5,7,8,10,13] and take on greater significance when the analysis is extended to the realities of low- and middle-income countries, where healthcare facilities often lack, among other things, basic water services [1].

Beyond healthcare professionals’ recognition that they have a role in minimizing the risk of HAIs to patients and themselves, a significant factor identified is the partnership between healthcare professionals and patients concerning hand hygiene [30]. The engagement between professionals and patients has resulted in more sustainable changes and increased adherence, mainly if healthcare workers facilitate patient hand hygiene and potentiate hygiene compliance [28].

A first comprehensive overview of these findings recognizes the presence of particularly relevant stressors, which aligns with Neuman’s model [18,19]. A multifaceted array of factors influences patients’ and healthcare professionals’ decision making, encompassing cognitive understanding of IPC measures, emotional responses to environmental conditions, and communication barriers between healthcare professionals, patients, and families. As Butenko et al. assert, the mere intention to discuss and collaborate does not guarantee translation into action, underscoring these factors’ complexity and multifaceted nature [30].

Other stressors [18,19] of high importance include variability in professional adherence to protocols and guidelines, inconsistent training and lack of knowledge, as identified in our findings [28,30]. Although multidisciplinary interventions and education have been implemented with proven results [28,42], it is essential to note that education alone is rarely sufficient to influence long-term behavioral change [42]. Again, these findings are consistent with the proposed multimodal approach for IPC [1,3,4,5,7,8,10,13]. However, despite the existence and availability of the recommendations, a 2024 ECDC Report [47] highlights differences in the level of training and skills of healthcare workers applying the definitions and differences in reporting behavior between hospitals and countries. In line with this report, our findings suggest investment in trained healthcare staff, informative documentation for patients and staff, posters and educational videos [30]. The efficacy of structured educational programs for healthcare professionals and patients has been demonstrated at all levels of prevention [1,4,5,6,8,12,13,14,16,48,49].

The organization given to the presentation and discussion of the results obtained with this umbrella review begins with the standard measures due to their undeniable importance in preventing the transmission of resistant organisms to patients and healthcare providers and ensuing contributions to HAI prevention and control [1,3,4,5,7,8,10,13,16].

Subsequently, it is essential to discuss the results associated with the CDC categorization of HAIs [10]. The results obtained are specific nursing interventions focused on preventing HAIs, namely aiming to preserve the integrity of the patient’s system by extending the flexible line of defense, including risk factor recognition, and overlapping with primary response interventions [18,19].

This can be seen in the use of skin disinfection solutions before vascular catheter insertion [42] and staff education for infection control programs [26,28,30,42,49], the use of chlorhexidine- or other antiseptic agent-impregnated dressing for vascular catheters [32,38] and taurolidine solutions to lock catheters [40,46] or in the proposal of performing device fixation without sutures [32] for preventing catheter-related bloodstream infection interventions that globally demonstrate their effectiveness [50,51]. In the context of surgical site infection prevention, primary strategies include a nursing assessment of the patient [41], skin preparation using antiseptics [33], e.g., chlorhexidine [25], antibiotic prophylaxis [41], the prevention of hypothermia by warming the patient and temperature management [39,43], and perioperative high-inspiration oxygen [39]. These interventions have been identified as evidence-based practices [52,53,54,55], and their efficacy is further enhanced by other interventions not revealed by this review, namely the administration of topical vancomycin powder and the irrigation of the surgical site with a povidone–iodine solution prior to closure [54], the optimization of nutrition, the prevention of anemia, and the promotion of early mobilization and preparation for discharge have been recognized as crucial components of the intervention [52,53,54,55]. Positioning ventilated patients [44] with an elevated headboard (30° to 60°), performing oral or hygiene care with chlorhexidine, both as a mouth rinse or in gel form [34], and using a silver-coated endotracheal tube [35] are identified as effective interventions in line with the results of other studies [56,57,58,59,60]. For catheter-associated urinary tract infection prevention, our results identify the insertion of the catheters by adequately trained people, removing them as quickly as possible, with late-night catheter removal decreasing the risk of catheter reinsertion when compared to early-morning removal [36].

Globally, these results are in conformity with the guidelines and orientation available for best practices [1,3,4,5,7,8,10,13,61] and highlight the importance of early identification and anticipation of the patient’s reaction to the environment, potentially infectious where HAIs are concerned, and implementing evidence-based nursing interventions potentially improves the client system in all domains, thereby contributing to the best response to the stressors [18,19].

This umbrella review also provides secondary interventions, actions that reinforce the defense lines, namely the daily assessment of the insertion site for localized infection or whenever a patient shows symptoms suggestive of infection [36].

In closing this discussion, beyond the identification of nurse-performed interventions that were systematized through this umbrella review that included systematic reviews, some with metanalysis, it was possible to survey this multifaceted challenge of HAI prevention, confronting the findings with the existing guidelines from international organizations that can support this practice worldwide, using the comprehensive perspective of Neuman’s Systems Model [18,19,62].

### Study Limitations

Umbrella reviews aim to review existing reviews. The initial challenge is that certain studies pertinent to the investigation may not have been incorporated due to variations in nurses’ core competencies across different countries. This prompted comprehensive discussion with all authors to reach a consensus. Despite the team’s best efforts, it is not possible to guarantee that any of the studies were not unduly excluded. Though the quality of the studies included in this review is high, the certainty of the evidence, and consequently the effect estimate, is moderate, essentially due to inconsistency and significant heterogeneity, namely, small, non-homogeneous samples in most studies with a high risk of bias. The lack of longitudinal data restricts understanding of long-term outcomes, while heterogeneity in intervention protocols and inconsistent outcome measures complicate comparisons across studies. The limited generalizability of findings due to unrepresentative study populations further weakens the external validity. These limitations underscore the need for well-designed, large-scale, randomized clinical trials with standardized methodologies to confirm and extend current findings. Undeniably, however, our umbrella review presents a unique synthesis of the evidence on HAI preventive interventions that will be of benefit to interested readers.

## 5. Conclusions

This umbrella review aimed to contribute to one of the most pressing needs worldwide, the prevention of HAIs, one of the leading causes of morbidity and mortality. By synthesizing the currently nurse-performed approaches to preventing HAIs and emphasizing their effectiveness, despite the identified limitations, this review highlights the importance of evidence-based practices for preventing HAIs.

Nursing interventions show varied effectiveness in preventing HAIs. Chlorhexidine use and taurolidine locks reduce infections with moderate certainty. Perioperative measures and ventilator-specific interventions offer small to moderate effects. Education, hand hygiene, and standardized care moderately improve outcomes. While robust primary studies are still needed, the integrated findings offer valuable insights into improving HAI prevention enriched by Neuman’s system model analysis. From this perspective, the importance of overseeing this issue with the aim of identifying and reducing stressors (or the potential for stressors) leads to the integration of primary and secondary prevention interventions to maintain, achieve or sustain an optimal level of well-being for the patient, understanding the impact not only of the stressor but also of the response of the system.

Adapting these practices and perspectives to specific contexts can be challenging. The main conclusion this analysis provides is the priority of continuing to invest in education, at all levels, from professionals, patients, families and communities, actively promoting sustainable, innovative nursing practices and sharing evidence-based practices locally, nationally, and internationally, particularly in low- and middle-income countries, building bridges across all health system levels.

## Figures and Tables

**Table 1:** Standard measures to prevent HAIs.

Interventions to Improve Patient Hand Hygiene			
Outcome Measures	Impact	N° of Participants (Studies)	Certainty of the Evidence (GRADE)
Incidence of HAIs	Effective improvement with moderate positive effects **	Participants: N/A 10 studies	⨁⨁⨁◯ MODERATE due to inconsistency (due to significant heterogeneity)

** The effect is interpreted as moderate positive because it is greater than 0.40 and less than 0.80. N/A—Not Available. GRADE Working Group grades of evidence: High certainty: We are very confident that the true effect lies close to that of the estimate of the effect. Moderate certainty: We are moderately confident in the effect estimate: The true effect is likely to be close to the estimate of the effect, but there is a possibility that it is substantially different. Low certainty: Our confidence in the effect estimate is limited: The true effect may be substantially different from the estimate of the effect. Very low certainty: We have very little confidence in the effect estimate: The true effect is likely to be substantially different from the estimate of effect.

**Table 2:** Interventions for preventing catheter-related bloodstream infections.

**Central line**	**Medication-Impregnated Dressing Products**			
	Outcome Measures	Impact	N° of Participants (Studies)	Certainty of the Evidence (GRADE)
	Incidence of catheter-related bloodstream infections	Effective improvement with moderate positive effects **	7436 participants (22 studies)	⨁⨁⨁◯ MODERATE due to inconsistency (due to significant heterogeneity)
	**Dressing Changes**			
	Outcome Measures	Impact	N° of Participants (Studies)	Certainty of the Evidence (GRADE)
	Frequency of CVAD dressing changes on the incidence of catheter-related infections	Effective improvement with small positive effects *	2277 participants (5 studies)	(⊕◯◯◯) VERY LOW
	**Use Taurolidine Lock Solutions**			
	Outcome Measures	Impact	N° of Participants (Studies)	Certainty of the Evidence (GRADE)
	The time interval from start of locking and infection	Effective improvement with moderate positive effects **	476 participants (4 studies)	⨁⨁⨁◯ MODERATE due to inconsistency (due to significant heterogeneity)
	Incidence rate of bloodstream infections	Effective improvement with moderate positive effects **	907 participants (10 studies)	⨁⨁⨁◯ MODERATE due to inconsistency (due to significant heterogeneity)
	Number of catheter removals due to infection;	Effective improvement with moderate positive effects **	476 participants (4 studies)	⨁⨁⨁◯ MODERATE due to inconsistency (due to significant heterogeneity)
	Catheter thrombosis	Effective improvement with moderate positive effects **	476 participants (4 studies)	⨁⨁⨁◯ MODERATE due to inconsistency (due to significant heterogeneity)
	**Silver-Impregnated Implantable Collagen Cuffs Use in Combination with a Specific Catheter Removal Policy. Interventions of Education and Training, a Standardized Catheter Care Protocol**			
	Outcome Measures	Impact	N° of Participants (Studies)	Certainty of the Evidence (GRADE)
	Incidence of catheter-related bloodstream infections	Effective improvement with moderate positive effects **	Participants: N/A (23 studies)	⨁⨁⨁◯ MODERATE due to inconsistency (due to significant heterogeneity)
	**Chlorhexidine-Impregnated Dressing on the Risk of Vascular and Epidural Catheter Bacterial Colonization and Infection**			
	**Outcome Measures**	**Impact**	**N° of Participants** **(Studies)**	**Certainty of the Evidence (GRADE)**
	Proportion of patients with either exit-site or catheter colonized with bacteria and systemic infections such as bloodstream	Effective improvement with moderate positive effects **	2396 participants (8 studies)	⨁⨁⨁◯ MODERATE due to inconsistency (due to significant heterogeneity)
	CNS infection related to a vascular catheter and an epidural catheter	Effective improvement with moderate positive effects **	2396 participants (8 studies)	⨁⨁⨁◯ MODERATE due to inconsistency (due to significant heterogeneity)
	Number of colonized epidural catheters when they were removed	Effective improvement with moderate positive effects **	2396 participants (8 studies)	⨁⨁⨁◯ MODERATE due to inconsistency (due to significant heterogeneity)
	Risk of complications by SPCs	Effective improvement with moderate positive effects **	7323 participants (24 studies)	⨁⨁⨁◯ MODERATE due to inconsistency (due to significant heterogeneity)
**Peripheral line**	**Use of Short-Term Peripheral Venous Catheters**			
	Outcome Measures	Impact	N° of Participants (Studies)	Certainty of the Evidence (GRADE)
	Incidence of bloodstream infections	Effective improvement with moderate positive effects **	85,063 participants (63 studies)	⨁⨁⨁◯ MODERATE due to inconsistency (due to significant heterogeneity)
	Impact of PVC duration on the risk of catheter colonization	Effective improvement with moderate positive effects **	85,063 participants (63 studies)	⨁⨁⨁◯ MODERATE due to inconsistency (due to significant heterogeneity)
	Effectiveness of central venous catheters treated with anti-infective agents	Effective improvement with moderate positive effects **	8655 participants (38 studies)	⨁⨁⨁◯ MODERATE due to inconsistency (due to significant heterogeneity)
	**Replacement of PIVC**			
	Outcome Measures	Impact	N° of Participants (Studies)	Certainty of the Evidence (GRADE)
	Assessed catheter-related bloodstream infection	Effective improvement with small positive effects *	7412 participants (9 studies)	⨁⨁⨁◯ MODERATE due to inconsistency (due to significant heterogeneity)
	Catheter-related bloodstream infection incidence	Effective improvement with small positive effects *	7412 participants (9 studies)	⨁⨁⨁◯ MODERATE due to inconsistency (due to significant heterogeneity)

* The effect is interpreted as small positive because it is less than 0.40. ** The effect is interpreted as moderate positive because it is greater than 0.40 and less than 0.80. N/A—Not Available. GRADE Working Group grades of evidence: High certainty: We are very confident that the true effect lies close to that of the estimate of the effect. Moderate certainty: We are moderately confident in the effect estimate: The true effect is likely to be close to the estimate of the effect, but there is a possibility that it is substantially different. Low certainty: Our confidence in the effect estimate is limited: The true effect may be substantially different from the estimate of the effect. Very low certainty: We have very little confidence in the effect estimate: The true effect is likely to be substantially different from the estimate of effect.

**Table 3:** Interventions to prevent Surgical Site Infections.

Use of Chlorhexidine with the Use of Iodine for Preoperative Skin Antisepsis			
Outcome Measures	Impact	N° of Participants (Studies)	Certainty of the Evidence (GRADE)
Risk of epidural	Effective improvement with moderate positive effects **	2396 participants (8 studies)	⨁⨁⨁◯ MODERATE due to inconsistency (due to significant heterogeneity)
Intravascular catheter or exit-site bacterial colonization	Effective improvement with moderate positive effects **	2396 participants (8 studies)	⨁⨁⨁◯ MODERATE due to inconsistency (due to significant heterogeneity)
Trend toward reduction in catheter-related bloodstream or CNS infections	Effective improvement with moderate positive effects **	2396 participants (8 studies)	⨁⨁⨁◯ MODERATE due to inconsistency (due to significant heterogeneity)
**Daily Bathing with Chlorhexidine**			
Outcome Measures	Impact	N° of Participants (Studies)	Certainty of the Evidence (GRADE)
Diagnostic criteria for BSIs, form and concentration of topical CHG	Effective improvement with moderate positive effects **	848 participants (12 studies)	⊕⊕⊕⊕ HIGH
Incidence of BSIs	Effective improvement with moderate positive effects **	848 participants (12 studies)	⊕⊕⊕⊕ HIGH
**Use the Perioperative High Oxygen Therapy**			
Outcome Measures	Impact	N° of Participants (Studies)	Certainty of the Evidence (GRADE)
Hyperoxia compared with low oxygen or controls in the prevention of surgical site infection	Effective improvement with small positive effects *	2728 participants (7 studies)	⊕⊕OO. LOW
**Administration of Antibiotic Prophylaxis (Surgical Site Infections Category)**			
Outcome Measures	Impact	N° of Participants (Studies)	Certainty of the Evidence (GRADE)
Incidence of surgical site infection after the administration of antibiotic prophylaxis within different timing intervals from the first incision in non-clean and implant surgical procedures	Effective improvement with small positive effects *	54,552 patients (21 studies)	⊕⊕OO. LOW
**Perioperative Warming (Surgical Site Infections Category)**			
Outcome Measures	Impact	N° of Participants (Studies)	Certainty of the Evidence (GRADE)
Occurrence of SSI	Effective improvement with small positive effects *	1465 participants (9 studies)	⨁⨁⨁◯ MODERATE due to inconsistency (due to significant heterogeneity)

* The effect is interpreted as small positive because it is less than 0.40. ** The effect is interpreted as moderate positive because it is greater than 0.40 and less than 0.80. N/A—Not Available. GRADE Working Group grades of evidence: High certainty: We are very confident that the true effect lies close to that of the estimate of the effect. Moderate certainty: We are moderately confident in the effect estimate: The true effect is likely to be close to the estimate of the effect, but there is a possibility that it is substantially different. Low certainty: Our confidence in the effect estimate is limited: The true effect may be substantially different from the estimate of the effect. Very low certainty: We have very little confidence in the effect estimate: The true effect is likely to be substantially different from the estimate of effect.

**Table 4:** Interventions to prevent Ventilation-Associated Pneumonia (VAP).

Semi-Recumbent Positioning Versus Supine Positioning			
**Outcome Measures**	**Impact**	**N° of Participants** **(Studies)**	**Certainty of the Evidence (GRADE)**
Clinically suspected VAP Microbiologically confirmed VAP Follow-up > 48 h	Effective improvement with small positive effects *	878 participants (10 studies)	⊕⊕OO LOW
**Oral Hygiene Care**			
**Outcome Measures**	**Impact**	**N° of Participants** **(Studies)**	**Certainty of the Evidence (GRADE)**
Effects of oral hygiene care on incidence of ventilator-associated pneumonia in critically ill patients receiving mechanical ventilation in hospital intensive care units (ICUs).	Effective improvement with moderate positive effects **	6016 participants (38 studies)	⨁⨁⨁◯ MODERATE due to inconsistency (due to significant heterogeneity)
**Silver-Coated ETT**			
**Outcome Measures**	**Impact**	**N° of Participants** **(Studies)**	**Certainty of the Evidence (GRADE)**
Risk of VAP at any time in participants intubated for ≥24 h	Effective improvement with moderate positive effects **	2081 participants (3 studies)	⨁⨁⨁◯ MODERATE due to inconsistency (due to significant heterogeneity)
Hospital mortality	Effective improvement with moderate positive effects **	2081 participants (3 studies)	⨁⨁⨁◯ MODERATE due to inconsistency (due to significant heterogeneity)
Device-related adverse events	Effective improvement with moderate positive effects **	2081 participants (3 studies)	⨁⨁⨁◯ MODERATE due to inconsistency (due to significant heterogeneity)

* The effect is interpreted as small positive because it is less than 0.40. ** The effect is interpreted as moderate positive because it is greater than 0.40 and less than 0.80. N/A—Not Available. GRADE Working Group grades of evidence: High certainty: We are very confident that the true effect lies close to that of the estimate of the effect. Moderate certainty: We are moderately confident in the effect estimate: The true effect is likely to be close to the estimate of the effect, but there is a possibility that it is substantially different. Low certainty: Our confidence in the effect estimate is limited: The true effect may be substantially different from the estimate of the effect. Very low certainty: We have very little confidence in the effect estimate: The true effect is likely to be substantially different from the estimate of effect.

**Table 5:** Interventions to prevent catheter-associated urinary tract infections.

Intervention: Removal of Short-Term Indwelling Urethral Catheterisation			
Outcome Measures	Impact	N° of Participants (Studies)	Certainty of the Evidence (GRADE)
Number of participants requiring recatheterisation; Symptomatic catheter-associated urinary tract infection (CAUTI); Dysuria; Condition-specific QoL or generic QoL measure	Effective improvement with small positive effects *	12,241 participants (91 studies)	⨁⨁⨁◯ MODERATE due to inconsistency (due to significant heterogeneity)

* The effect is interpreted as small positive because it is less than 0.40. GRADE Working Group grades of evidence: High certainty: We are very confident that the true effect lies close to that of the estimate of the effect. Moderate certainty: We are moderately confident in the effect estimate: The true effect is likely to be close to the estimate of the effect, but there is a possibility that it is substantially different. Low certainty: Our confidence in the effect estimate is limited: The true effect may be substantially different from the estimate of the effect. Very low certainty: We have very little confidence in the effect estimate: The true effect is likely to be substantially different from the estimate of effect.

## Data Availability

Data are reported in the current study.

## Supplementary Materials

The following supporting information can be downloaded at: https://www.mdpi.com/article/10.3390/microorganisms13020463/s1, Table S1: Quality Assessment; Table S2: Summary of Findings organized according to CDC’s categories; Appendix SA.1: Search Strategy; Appendix SA.2: Table with the Characteristics of the Studies.
