# Supplementary material for: Current Approaches on Nurse-Performed Interventions to Prevent Healthcare-Acquired Infections: An Umbrella Review
Source: Microorganisms. 2025 Feb 19;13(2):463. doi: 10.3390/microorganisms13020463 (PMC11858086; doi:10.3390/microorganisms13020463)
Supplement: Supplementary file 1 [file microorganisms-13-00463-s001.zip › Tables S1 and S2.pdf]

microorganisms

MDPI

Table S1. Quality assessment.

Critical Appraisal for Systematic Reviews													Quality of the evidence
**Authors│Year**	**1**	**2**	**3**	**4**	**5**	**6**	**7**	**8**	**9**	**10**	**11**	**Quality**	
Ho and Litton, 2006	N	Y	Y	Y	Y	Y	N	Y	Y	Y	Y	82% Moderate	1 a.
Ramritu et al., 2008	Y	Y	Y	Y	Y	Y	Y	Y	Y	Y	Y	100% High	1.a.
Hockenhull et al., 2009	Y	N	N	Y	Y	Y	Y	Y	N	Y	Y	73% Moderate	1 b.
Lee et al., 2010	Y	Y	Y	N	Y	N	Y	Y	Y	Y	Y	82% Moderate	1 b.
O'Horo et al., 2012	Y	Y	Y	Y	Y	Y	Y	Y	Y	Y	Y	100% High	1 a.
Togioka et al., 2012	Y	Y	Y	Y	Y	Y	Y	Y	Y	Y	Y	100% High	1.a.
Liu et al., 2013	N	Y	Y	Y	Y	Y	N	Y	Y	N	Y	73% Moderate	1 a.
Ullman et al., 2015	Y	Y	Y	Y	Y	Y	Y	Y	Y	Y	Y	100% High	1.b.
Tokmaji et al., 2015	Y	Y	Y	Y	Y	Y	Y	Y	Y	Y	Y	100% High	1 b.
Wang et al., 2016	Y	Y	Y	Y	Y	Y	Y	Y	Y	Y	Y	100% High	1 a.
Srigley, Furness and Gardam, 2016	Y	Y	Y	Y	Y	Y	Y	Y	Y	Y	Y	100% High	1 b.
Gavin et al., 2016	Y	Y	Y	Y	Y	Y	Y	Y	Y	Y	Y	100% High	1 b.
Hua et al., 2016	Y	Y	Y	Y	Y	Y	Y	Y	Y	Y	Y	100% High	1 b.
Butenko, Lockwood and McArthur, 2017	Y	Y	Y	Y	Y	Y	Y	Y	Y	Y	Y	100% High	1 b.
Mermel, 2017	Y	Y	Y	Y	N	N	Y	Y	N	Y	N	36% Low	1 b.
Jonge et al., 2017	Y	Y	Y	Y	Y	Y	Y	Y	N	Y	Y	91% High	1 a.
Webster et al., 2019	Y	Y	Y	Y	Y	Y	Y	Y	Y	Y	Y	100% High	1 b.
Hopkinson et al., 2020	Y	U	Y	Y	Y	Y	Y	U	N	Y	Y	73% Moderate	1 b.
Poveda, Oliveira and Galvão, 2020	Y	Y	Y	Y	Y	Y	Y	Y	N	Y	Y	91% High	1.a
Sun, Wan and Liang, 2020	Y	Y	Y	Y	Y	Y	Y	Y	N	Y	Y	91% High	1 a.
Ellahi et al., 2021	Y	Y	Y	Y	Y	Y	Y	Y	Y	Y	Y	100% High	1 b.
Nuckols et al., 2016	Y	Y	Y	Y	Y	Y	Y	Y	Y	Y	Y	100% High	1.b.
% (within the indicator)	91	91	96	96	96	91	91	96	73	96	96		
Legend: Y—Yes, N—No.													

Table S2. Summary of findings organized according to CDC’s categories.

	2006	2008	2009	2010	2012		2013	2015		2016				2017			2019	2020				2021
	Ho and Litton,	Ramritu et al.	Hockenhull et al.	Lee et al.	O’ Horo et al.	Togioka et al.	Liu et al.	Ullman et al.	Tokmaji et al.	Wang et al.	Srigley et al.	Gavin et al.	Nuckols et al.	Butenko et al.	Mermel	Jonge et al.	Webster et al.	Hua et al.	Hopkinson et al.	Poveda et al.	Sun et al.	Ellahi et al.
SM		x			x						x	x		x								
SSI				x		x					x					x				x		
CRBSI	x	x	x		x		x	x				x	x		x		x		x		x	
VAP									x	x								x				
CAUTI																						x

Legend: SM: standard measure; SSIs: surgical site infections; CRBSI: catheter-related bloodstream infection; VAP: ventilation-associated pneumonia; CAUTI: catheter-associated urinary tract infection.
