# Supplementary material for: Current Approaches on Nurse-Performed Interventions to Prevent Healthcare-Acquired Infections: An Umbrella Review
Source: Microorganisms. 2025 Feb 19;13(2):463. doi: 10.3390/microorganisms13020463 (PMC11858086; doi:10.3390/microorganisms13020463)
Supplement: Supplementary file 1 [file microorganisms-13-00463-s001.zip › Appendix SA.1.pdf]

Appendix SA.1 – Search Strategy

Databases	Search Stragegy
CINAHL Complete, MEDLINE Complete, Cochrane Central Register of Controlled Trials (via EBSCOhost)	*(((“acute patient” OR (MM "Inpatients") OR (MM "Patients+") OR “acute”)) AND ((MM "Pneumonia, Ventilator-Associated") OR (MM "Healthcare-Associated Pneumonia") OR "intubation-associated pneumonia" OR "surgical site infection" OR (MM "Urinary Tract Infections, Catheter-Related") OR (MM "Catheter-Related Infections+") OR (MM "Catheter-Related Bloodstream Infections") OR “peripheral vascular catheter” OR “central vascular catheter”) AND ("interventions" OR "bundle" OR (MM "Practice Guidelines") OR "guidelines")) AND (("healthcare-associated infections") OR (MM "Incidence") OR (MM "Prevalence") OR (MM "Mortality+") OR (MM "Length of Stay") OR "prevention" OR "control")))*
Cochrane Database of Systematic Reviews (via EBSCOhost)	*(((“acute patient” OR inpatients OR patients OR acute)) AND (("pneumonia, ventilator-associated" OR "healthcare-associated Pneumonia" OR "intubation-associated pneumonia" OR "surgical site infection" OR “urinary tract Infections, catheter-related" OR “catheter-related infections" OR “catheter-related bloodstream Infections" OR “peripheral vascular catheter” OR “central vascular catheter”) AND (interventions OR bundle OR “practice guidelines” OR guidelines)) AND (("healthcare-associated infections" OR incidence OR prevalence OR mortality OR “length of stay” OR prevention OR control)))*
JBI EBP Resources on Ovid	*((acute or inpatients or patients) and ((pneumonia, ventilator-associated or healthcare-associated Pneumonia or intubation-associated pneumonia or surgical site infection or urinary tract infections, catheter-related or catheter-related infections or catheter-related bloodstream Infections or peripheral vascular catheter or central vascular catheter) and (interventions or bundle or practice guidelines or guidelines)) and (healthcare-associated infections or incidence or prevalence or mortality or length of stay or prevention or control))*
Scopus	*ABS((acute OR "acute patient") AND (healthcare-associated infections) AND (safety))*
Web of Science	*((AB=(healthcare-associated infections)) AND AB=(acute patient*)) AND AB=(prevent*)*
Google Scholar	*(((“acute patient” OR inpatients OR patients OR acute)) AND (("pneumonia, ventilator-associated" OR "healthcare-associated Pneumonia" OR "intubation-associated pneumonia" OR "surgical site infection" OR “urinary tract Infections, catheter-related" OR “catheter-related infections" OR “catheter-related bloodstream Infections" OR “peripheral vascular catheter” OR “central vascular catheter”) AND (interventions OR bundle OR “practice guidelines” OR guidelines)) AND (("healthcare-associated infections" OR incidence OR prevalence OR mortality OR “length of stay” OR prevention OR control) AND (systematic review OR metanalysis)))*
