# Supplementary material for: Current Approaches on Nurse-Performed Interventions to Prevent Healthcare-Acquired Infections: An Umbrella Review
Source: Microorganisms. 2025 Feb 19;13(2):463. doi: 10.3390/microorganisms13020463 (PMC11858086; doi:10.3390/microorganisms13020463)
Supplement: Supplementary file 1 [file microorganisms-13-00463-s001.zip › Appendix SA.2.pdf]

# Appendix SA.2 – Table with the Characteristics of the studies

| Studies | Author/Year/Location | Study design | Aim | Study Population | Outcome measures | Types of interventions | Results |
|---|---|---|---|---|---|---|---|
| Use of chlorhexidine-impregnated dressing to prevent vascular and epidural catheter colonization and infection: a meta-analysis | (Ho & Litton, 2006) Australia | Systematic Review and Meta - analysis | To assess the effect of chlorhexidine-impregnated dressing on the risk of vascular and epidural catheter bacterial colonization and infection | 2396 participants (8 studies*) | Proportion of patients with either exit-site or catheter colonized with bacteria and systemic infections such as bloodstream and central nervous system infections (CNS) infection related to a vascular catheter and an epidural catheter, respectively. Number of colonized epidural catheters when they were removed. | Chlorhexidine-impregnated dressing on the risk of vascular and epidural catheter bacterial colonization and infection (Central line-associated Bloodstream infections category) | The use of a chlorhexidine-impregnated dressing has been associated with a tendency toward reduced catheter-related blood flow or CNS infections. Local cutaneous reactions to chlorhexidine-impregnated dressings were reported in 5.6% of patients in three studies and 96% of these reactions occurred in neonatal patients. The number needed to prevent one episode of intravascular catheter-related bloodstream infection was 142 for an average catheter in situ period of 10 days and dressing changes every 5 days. The chlorhexidine-impregnated dressing is effective in reducing bacterial colonization of the vascular and epidural catheter and is also associated with a tendency toward reduced catheter-related bloodstream or CNS infections. |
| Systematic review and cost analysis comparing use of chlorhexidine with use of iodine for preoperative skin antisepsis to prevent surgical site infection | (Lee et al., 2010) USA | Systematic Review | To compare the use of chlorhexidine with the use of iodine for preoperative skin antisepsis with respect to effectiveness in preventing surgical site infections (SSIs) and cost. | 3614 participants (9 RCT) | Effectiveness in preventing surgical site infections (SSIs) and cost. | Use of chlorhexidine with the use of iodine for preoperative skin antisepsis (Surgical site infections category) | Chlorhexidine antisepsis was associated with significantly fewer SSIs and positive skin culture results than iodine antisepsis. Sensitivity analyses showed that net cost savings persisted under most circumstances. Preoperative skin antisepsis with chlorhexidine is more effective than preoperative skin antisepsis with iodine for preventing SSI and results in cost savings. |
| The role of perioperative high inspired oxygen therapy in reducing surgical site infection: a meta-analysis | (Togioka et al., 2012) USA | Systematic review and meta - analysis | To determine whether perioperative hyperoxia reduces surgical site infection | 2728 participants (7 RCT) | Evaluation of the role of hyperoxia compared with low oxygen or controls in the prevention of surgical site infection | Use the perioperative high oxygen therapy (Surgical site infections category) | Perioperative high-inspired oxygen therapy overall was not found to be beneficial for preventing surgical site infection based on this meta-analysis. The positive results of 2 subgroup analyses (general anesthesia and colorectal surgery trials) suggest a benefit for hyperoxia in decreasing surgical site infection. A meta-analysis of all trials that met inclusion criteria did not show that high inspired perioperative oxygen therapy is beneficial for preventing surgical site infections. |
| Taurolidine lock solutions for the prevention of catheter-related bloodstream infections: a systematic review and meta-analysis of randomized controlled trials | (Liu et al., 2013) China | Systematic Review and Meta - analysis | To assess the efficacy of taurolidine lock solutions (TLS) for preventing: Catheter-related bloodstream infections (CRBSIs). | 431 participants (6 RCT) | Incidence of CRBSIs | Use taurolidine lock solutions for preventing: Catheter-related bloodstream infections (CRBSIs) (Central line-associated Bloodstream infections category) | The use of TLS reduced the incidence of CRBSIs without obvious adverse effects or bacterial resistance. However, the susceptibility of G+ and G- bacteria to taurolidine and the risk for catheter-associated thrombosis of TLS are indeterminate due to limited data. The results should be treated with caution due to the limited sample sizes and methodological deficiencies of the included studies. |
| Short-term peripheral venous catheter "related bloodstream infections: a systematic review | (Mermel, 2017) USA | Systematic review | To determine the magnitude of bloodstream infections (BSIs) related to the use of Short-term peripheral venous catheters (PVCs) | 85063 participants (11 RCT) | Incidence of PVCR-BSI involving, impact of PVC duration on the risk of catheter colonization | Use of Short-term peripheral venous catheters (PVCs) (Central line-associated Bloodstream infections category) | Blood cultures should be obtained in patients with evidence of PVC infection and systemic symptomatology such as fever, carefully inspect the PVC insertion site in bacteremic or fungemic patients, and remove PVCs associated with localized infection with or without associated BSI. |
| Timing of preoperative antibiotic prophylaxis in 54,552 patients and the risk of surgical site infection: A systematic review and meta-analysis | (Jonge et al., 2017) USA | Systematic Review and Meta - analysis | To assess the effect of the timing of preoperative surgical antibiotic prophylaxis (SAP) on surgical site infection (SSI) rates and compare different timing intervals. The study aimed to provide evidence for the development of recommendations for the World Health Organization guideline for SSI prevention. | 54 552 participants (22 studies*) | Incidence of surgical site infection after the administration of antibiotic prophylaxis within different timing intervals from the first incision in non-clean and implant surgical procedures | Administration of antibiotic prophylaxis (Surgical site infections category) | Administration of antibiotic prophylaxis more than 120 minutes before or after incision is associated with a higher risk of surgical site infections compared to administration within 120 minutes before incision. No clear advantage was found within the 120-minute window before incision, contrary to the commonly accepted practice of a 60-minute window for prophylaxis administration. Surgical antibiotic prophylaxis should be administered within 120 minutes prior to incision, when indicated according to the type of operation. Administration before 120 minutes or after incision is associated with a higher risk of surgical site infection. The exact optimal timing within this timeframe cannot be defined according to the available evidence but half-life and protein binding of the antibiotic should be taken into account, also according to the underlying conditions of the individual patient. The broadly accepted recommendation to administer AP within 60 minutes prior to incision could not be substantiated. |
| Catheter-related bloodstream infections in intensive care units: a systematic review with meta-analysis | (Ramritu et al., 2008) Australia | Systematic Review and Meta-Analysis | To evaluate strategies, other than antimicrobial coated catheters, hypothesized to reduce risk of CRBSI and catheter colonization in the ICU setting. | Number of participants is not provided. (23 studies*) | Incidence of CRBSI | Using silver-impregnated implantable collagen cuffs in combination with a specific catheter removal policy. Interventions of education and training, a standardized catheter care protocol (Central line-associated Bloodstream infections category) | A range of interventions may reduce risks of catheter-related bloodstream infection, in addition to antimicrobial catheters. Interventions other than antimicrobial catheters may be useful for reducing risks of CRBSI in the ICU setting. Infection control is typically nurse-led within a wider multidisciplinary team and nurses play a key role in the care of ICU patients, including assisting with the insertion and daily management of CVCs. Nurse awareness and implementation of appropriate risk reducing strategies is important. |
| Perioperative body temperature maintenance and occurrence of surgical site infection: A systematic review with meta-analysis. | (Poveda, Oliveira & Galvão, 2020) Brazil | A systematic review with meta-analysis | To assess the efficacy of active warming methods to maintain perioperative patients' body temperature and its effects on the occurrence of SSI. | 1465 (6 RCTs) | Occurrence of SSI | Perioperative warming (Surgical site infections category) | Perioperative body temperature maintenance has been associated with preventing surgical site infections. Active warming methods are effective in maintaining perioperative body temperature. The available scientific evidence does not clarify the benefits of warming methods in preventing surgical site infection. Current guidelines recommend perioperative warming as one of the strategies to prevent surgical site infection, although there are gaps in the knowledge produced on this issue. The generated evidence indicated that the use of an active warming method could maintain a higher average body temperature as well as decrease the incidence of surgical site infection. Exposure of the patient to temperatures below 36 °C in the perioperative period increased the chances of developing this type of infection. The meta-analysis indicated that the association between perioperative active warming methods compared with others to reduce the chances of developing surgical site infection remains unclear the employment of active warming methods may maintain higher body temperature. However, it is unclear if that method can prevent SSI. |

| | | | | | | | |
|---|---|---|---|---|---|---|---|
| Semi-recumbent position versus supine position for the prevention of ventilator-associated pneumonia in adults requiring mechanical ventilation. | (Wang et al., 2016) China | A systematic review with meta-analysis | To assess the effectiveness and safety of semi-recumbent positioning versus supine positioning to prevent ventilator associated pneumonia (VAP) in adults requiring mechanical ventilation. | 878 participants (10 RCTs) | Clinically suspected VAP microbiologically confirmed VAP Follow-up: > 48 hours<br>ICU mortality and Hospital mortality | Semi-recumbent positioning versus supine positioning<br>(Ventilator-associated Pneumonia - VAP) | A semi-recumbent position (30º to 60º) significantly reduced the risk of clinically suspected VAP compared to a 0º to 10º supine position. However, the evidence is seriously limited with a high risk of bias. No adequate evidence is available to draw any definitive conclusion on other outcomes and the comparison of alternative semi-recumbent positions. Adverse events, particularly venous thromboembolism, were under-reported. |
| Economic Evaluation of Quality Improvement Interventions for Bloodstream Infections Related to Central Catheters: A Systematic Review. | (Nuckols et al., 2016) USA | Systematic review | To systematically review economic evaluations of quality improvement (QI) interventions for the prevention of blood-stream infection related to the use of CVCs in the hospital setting,<br>considering both program costs and changes in infection-related costs. | Number of participants is not provided.<br>15 studies*, including data from 113 hospitals | Cost analysis<br>CEA<br>Health system,<br>Infection Related Costs<br>Incremental Net Cost<br>⊕⊕OO LOW | Interventions related to central venous catheters were: Checklists, simulation-based training, "time out," audit and feedback, checklists for CLABSI and VAP<br>(Central line-associated Bloodstream infections category and Ventilator-associated pneumonia - VAP) | Interventions related to central venous catheters were, on average, associated with 57% fewer bloodstream infections and substantial savings to hospitals.<br>Larger initial investments may be associated with greater savings.<br>Although checklists are now widely used and infections have started to decline, additional improvements and savings can occur at hospitals that have not yet attained very low infection rates.<br>Interventions designed to prevent CLABSI were, on average, associated with a 57% decline in infections as well as $1.85 million net savings to hospitals within 1 to 3 years, making them of high value to hospitals. Interventions that involve larger initial investments in resources may be associated with greater net savings.<br>Although checklists are now widely used and infection rates have declined, additional improvements and cost savings can occur at hospitals that have not yet attained very low infection rates. |
| Interventions to improve patient hand hygiene: a systematic review | (Srigley, Furness & Gardam, 2016) Canada | Systematic review | The primary objective was to determine the efficacy of patient hand hygiene interventions in<br>reducing HCAIs (e.g. Clostridium difficile infection) or AROs<br>[e.g. meticillin-resistant Staphylococcus aureus (MRSA)]<br>compared to usual care.<br>The secondary objective was to determine the efficacy of these interventions in improving patient hand hygiene rates | Number of participants is not provided.<br>(10 studies*) | Incidence of HCAIs | Interventions to improve patient hand hygiene may<br>(standard measures for prevent HAIs) | Interventions to improve patient hand hygiene may reduce the incidence of HCAIs and improve hand hygiene rates, but the quality of evidence is low. |
| The Efficacy of Daily Bathing with Chlorhexidine for Reducing Healthcare-Associated Bloodstream Infections: A Meta-analysis | (O'Horo et al., 2012) USA | Systematic Review and Meta-Analysis | To assess the efficacy of daily bathing with chlorhexidine (CHG) for prevention of healthcare-associated bloodstream infections (BSIs) | 137 392 patient-days (12 studies*) | Diagnostic criteria for BSIs<br>Form and concentration of topical CHG<br>Incidence of BSIs | Daily Bathing with Chlorhexidine<br>(surgical site infections) | Daily bathing with CHG reduced the incidence of BSIs, including central line-associated BSIs, among patients in the medical ICU. |
| Short Peripheral Catheter Dwell Time and Associated Complications: A Systematic Review. | (Hopkinson et al., 2020)<br>USA | Systematic review | To explore the current state of the science regarding Short Peripheral catheter dwell time as a predictor of (SPC) complications in adult inpatients.<br>To determine whether routine versus clinically indicated catheter replacement increased the risk of complications. | 7323 participants (17 studies*) | Risk of complicaton by SPCs | Catheter replacement:<br>Routine replacement vs clinically indicated replacement.<br>(Central line-associated Bloodstream infections category) | There is evidence that the risk of complications caused by short peripheral catheters (SPCs) does not increase when SPCs are replaced due to clinical indication versus at a specific time frame. In the studies, however, the dwell time does not typically exceed an average of 3.5 days.<br>It is uncertain how long SPCs may stay in place before there is an increased risk of complications or if there is an increased risk. |
| Patient experiences of partnering with healthcare professionals for hand hygiene compliance: A systematic review | (Butenko, Lockwood & McArthur, 2017) Australia | systematic review | To determine the best available evidence in relation to the experiences of the patient partnering with healthcare professionals for hand hygiene compliance. | 60 participants (3 studies*) | Inhibited by a self-perceived lack of knowledge and concerns about impact on care<br>Intention and action to speak up do not consistently occur and patents employ alternative tactics to partner for hand hygiene compliance | Hand hygiene compliance | The current review highlights the complexity of the patient's experience of partnering with healthcare professionals for hand hygiene compliance.<br>The experiences reported indicated that there is a possible disparity between the healthcare facility and healthcare professionals' promotion and intention of partnering for hand hygiene compliance, and the actual patient's acceptance, participation, partnership, experience and implementation of this initiative.<br>This disconnect between intent and action appears to be influenced by a number of factors including organizational structures as well as drivers such as cultural beliefs and behavior. The findings of this review indicate that the patient's experience relating to partnering for hygiene compliance with care professionals presents significant challenges, and the intention to speak and partner does not always translate into action.<br>Patients express varying levels of comfort regarding asking questions or challenging healthcare professionals.<br>The influencing reasons on the patient decision making process, and the lack of intention being followed by action are multi-factorial and steeped in both objective and subjective drivers of behavior. |
| Clinically indicated replacement versus routine replacement of peripheral venous catheters | (Webster et al., 2019) Australia | systematic review | To assess the effects of removing peripheral intravenous catheters when clinically indicated compared with removing and re-siting the catheter routinely. | 7412 participants (9 RCT's) | Assessed catheter-related bloodstream infection (CRBSI) incidence of thrombophlebitis | Routine replacement of PIVC<br>(Central line-associated Bloodstream infections category) | There is moderate-certainty evidence of no clear difference in rates of CRBSI, thrombophlebitis, all-cause BSI, mortality and pain between clinically indicated or routine replacement of PIVC. We are uncertain if local infection is reduced or increased when catheters are changed when clinically indicated. There is moderate-certainty evidence that infiltration and catheter blockage is probably lower when PIVC are changed routinely; and moderate-certainty evidence that clinically indicated removal probably reduces device-related costs. The addition of two new trials for this update found no further evidence to support changing catheters every 72 to 96 hours. Healthcare organisations may consider changing to a policy whereby catheters are changed only if there is a clinical indication to do so, for example, if there were signs of infection, blockage or infiltration. This would provide significant cost savings, spare patients the unnecessary pain of routine re-sites in the absence of clinical indications and would reduce time spent by busy clinicians on this intervention. |

| | | | | | | | |
|---|---|---|---|---|---|---|---|
| | | | | | | | To minimise PIVC-related complications, staff should inspect the insertion site at each shift change and remove the catheter if signs of inflammation, infiltration, occlusion, infection or blockage are present, or if the catheter is no longer needed for therapy |
| Dressings and securement devices for central venous catheters (CVC) | (Ullman et al., 2015) Australia | systematic review | To compare the available dressing and securement devices for CVCs, in terms of catheter-related bloodstream infection (BSI), catheter colonisation, entry- and exit-site infection, skin colonisation, skin irritation, failed catheter securement, dressing condition and mortality. | 7436 participants (22 studies*) | Incidence of catheter-related BSI | Medication-impregnated dressing products<br>(Central line-associated Bloodstream infections category) | Medication-impregnated dressing products reduce the incidence of catheter-related BSI relative to all other dressing types. There is some evidence that CGI dressings, relative to SPU dressings, reduce catheter-related BSI for the outcomes of frequency of infection per 1000 patient days, risk of catheter tip colonisation and possibly risk of catheter-related BSI.<br>A multiple treatment meta-analysis found that sutureless securement devices are likely to be the most effective at reducing catheter-related BSI though this is low quality evidence. |
| Frequency of dressing changes for central venous access devices on catheter-related infections | (Gavin et al., 2016) Australia | systematic review | To assess the effect of the frequency of CVAD dressing changes on the incidence of catheter-related infections and other outcomes including pain and skin damage. | 2277 participants (5 RCTs ) | Frequency of CVAD dressing changes on the incidence of catheter-related infections and other outcomes including pain and skin damage. | Dressing changes on the incidence of catheter-related<br>(Central line-associated Bloodstream infections category) | Repeated removal and application of dressings can cause damage to the skin. Less frequent dressing changes can reduce skin damage, but it is unclear whether this practice affects the frequency of catheter-related infections.<br>The best available evidence is currently inconclusive about whether longer intervals between CVAD dressing changes are associated with more or less catheter-related infection.<br>There is more evidence that CVAD dressings are associated with more or less catheter-related infection, mortality or pain than shorter intervals. |
| Oral hygiene care for critically ill patients to prevent ventilator-associated pneumonia | (Hua et al., 2020) China | systematic review | To assess the effects of oral hygiene care on incidence of ventilator-associated pneumonia in critically ill patients receiving mechanical ventilation in hospital intensive care units (ICUs). | 6016 participants (38 RCTs,) | Effects of oral hygiene care on incidence of ventilator-associated pneumonia in critically ill patients receiving mechanical ventilation in hospital intensive care units (ICUs). | Oral hygiene care<br>(Ventilator-associated Pneumonia - VAP) | OHC including chlorhexidine mouthwash or gel reduces the risk of developing ventilator-associated pneumonia in critically ill patients from 24% to about 18%. However, there is no evidence of a difference in the outcomes of mortality, duration of mechanical ventilation or duration of ICU stay.<br>There is no evidence that OHC including both antiseptics and toothbrushing is different from OHC with antiseptics alone, and some weak evidence to suggest that povidone iodine mouthrinse is more effective than saline/placebo, and saline rinse is more effective than saline swab in reducing VAP.<br>There is insufficient evidence to determine whether powered toothbrushing or other oral care solutions are effective in reducing VAP.<br>There is also insufficient evidence to determine whether any of the interventions evaluated in the studies are associated with adverse effects.<br>There is high quality evidence that chlorhexidine, either as a mouthrinse or a gel, reduces the risk of VAP from 25% to about 19%. |
| Silver-coated endotracheal tubes for prevention of ventilator-associated pneumonia in critically ill patients | (Tokmaji et al., 2015) Netherlands | systematic review | The primary objective was to investigate whether silver-coated ETTs are effective in reducing the risk of VAP and hospital mortality in comparison with standard non-coated ETTs in people who require mechanical ventilation for 24 hours or longer.<br>The secondary objective was to ascertain whether silver-coated ETTs are effective in reducing the following clinical outcomes: device-related adverse events, duration of intubation, length of hospital and intensive care unit (ICU) stay, costs, and time to VAP onset. | 2081 participants (3 RCTs) | Risk of VAP at any time in participants intubated for ≥ 24 hours<br>Hospital mortality<br>Device-related adverse events | Silver-coated ETT<br>(Ventilator-associated Pneumonia - VAP) | Silver-coated ETT seems to show a significant reduction with regards to the risk of VAP, especially during the first 10 days of mechanical ventilation in participants who require mechanical ventilation for 24 hours or longer.<br>This finding is of clinical importance because the peak time of a VAP event in mechanically ventilated patients is usually within 10 days after intubation. In addition, silver-coated ETT reduces time to VAP onset.<br>However, due to the low quality of evidence, it is possible that the findings of this review could merely be a reflection of random error.<br>Also, due to the fact that confidence intervals were insufficiently narrow to rule out an important magnitude of effect, there is a lack of evidence of effect regarding other important clinical outcomes, including hospital mortality, duration of intubation, and length of ICU and hospital stay. |
| Strategies for the removal of short-term indwelling urethral catheters in adults | (Ellahi et al., 2021) Australia | systematic review | To assess the effects of strategies for removing short-term (14 days or less) indwelling catheters in adults. | 12 241 participants (94 RCTs) | Number of participants requiring recatheterisation<br>Symptomatic catheter-associated urinary tract infection (CAUTI)<br>Dysuria<br>Condition-specific QoL or generic QoL measure | Removal of short-term indwelling urethral catheterization (volume of first void (mL), time to first void (hours), post-void residual volume (mL), length of hospitalisation (days), time between removal of catheter to discharge (days), health status/quality of life (condition-specific or generic quality-of-life measures, psychological outcome measures)<br>(Catheter-associated Urinary Tract Infections (CAUTI) category)." | There is some evidence to suggest the removal of indwelling urethral catheters late at night rather than early in the morning may reduce the number of people who require recatheterisation. It appears that catheter removal after shorter compared to longer durations probably reduces the risk of symptomatic CAUTI and may reduce the risk of dysuria. However, it may lead to more people requiring recatheterisation.<br>The other evidence relating to the risk of symptomatic CAUTI and dysuria is too uncertain to allow us to draw any conclusions.<br>Due to the low certainty of the majority of the evidence presented here, the results of further research are likely to change our findings and to have a further impact on clinical practice.<br>This systematic review has highlighted the need for a standardised set of core outcomes, which should be measured and reported by all future trials comparing strategies for the removal of short-term urinary catheters. |
| The clinical effectiveness of central venous catheters treated with anti-infective agents in preventing catheter-related bloodstream infections: a systematic review | (Hockenhull et al., 2009)<br>United Kingdom | systematic review | To assess the clinical effectiveness of central venous catheters (CVCs) treated with anti-infective agents (AI-CVCs) in preventing catheter-related bloodstream infections (CRBSI). | 8655 participants (38 studies*) | Effectiveness of central venous catheters (CVCs) treated with anti-infective agents (AI-CVCs) | Central venous catheters (CVCs) treated with antiseptic agents (AI-CVCs)<br>(Central line-associated Bloodstream infections category) | AI-CVCs appear to be effective in reducing CRBSI compared with standard CVCs.<br>However, it is important to establish whether this effect remains in settings where infection prevention bundles of care are established as routine practice |
| Taurolidine lock solution for catheter-related bloodstream infections in | (Sun, Wan and Liang, 2020)<br>China | Systematic Review and Meta-Analysis | To determine the efficacy of taurolidine lock solution in reducing catheter-related bloodstream infections | 431 participants (6 studies*) | Time interval from start of locking and infection<br>Incidence rate of BSI | Taurolidine lock solution for catheter-related bloodstream<br>(Central line-associated Bloodstream infections category) | Our results indicated a statistically significant reduction in the total number of CRBSI with taurolidine.<br>The number of catheters removed due to infection or suspected infection was not significantly different between the two groups.<br>The use of taurolidine as a catheter locking solution may significantly reduce CRBSI in pediatric patients. |

| | | | | | | | |
|---|---|---|---|---|---|---|---|
| pediatric patients: A meta-analysis | | | (CRBSI) in pediatric patients through a meta-analysis. | | Number of catheter removal due to infection<br>Catheter thrombosis | | |

*Type of study not available
